# Seven Years of 700 Cholesterol Without Coronary Atherosclerosis: A Lean Mass Hyper-Responder Case Report

**DOI:** 10.3390/diseases14050168

**Published:** 2026-05-11

**Authors:** Nicholas G. Norwitz, David Feldman, Adrian Soto-Mota

**Affiliations:** 1 Brigham and Women’s Hospital, Harvard Medical School, Boston, MA 02115, USA; 2 Citizen Science Foundation, Las Vegas, NV 89139, USA; dave@citizensciencefoundation.org; 3 Metabolic Diseases Research Unit, Instituto Nacional de Ciencias Médicas y Nutrición Salvador Zubirán, Mexico City 14080, Mexico; adrian.sotom@incmnsz.mx; 4 Tecnologico de Monterrey, School of Medicine, Mexico City 14380, Mexico

**Keywords:** ApoB, atherosclerosis, cholesterol, coronary CT angiography, ketogenic diet, Heartflow, lean mass hyper-responder

## Abstract

Background: While reducing LDL cholesterol (LDL-C) remains central focuses of conventional preventive cardiology, substantial heterogeneity exists in the cardiovascular risk associated with even extreme LDL-C elevations, likely depending heavily on the broader metabolic context. Specifically, the lean mass hyper-responder (LMHR) phenotype—characterized by markedly elevated LDL-C with elevated high-density lipoprotein cholesterol (HDL-C) and low triglycerides in the setting of a ketogenic diet—has recently been described, though its long-term risk profile remains poorly defined. Case Presentation: We describe a male in his 30s without any congenital dyslipidemia who adopted a ketogenic diet for the management of ulcerative colitis and who subsequently exhibited a sixfold increase in LDL-C from a baseline of 95 mg/dL to 574 mg/dL, with total cholesterol of up to 705 mg/dL, HDL-C at 124 mg/dL, and triglycerides at 34 mg/dL. Despite maintaining these extreme lipid levels for nearly seven years, he demonstrated no coronary plaque or stenosis on coronary computed tomography angiography (CCTA; CAD-RADS = 0). Additionally, quantification of coronary plaque as assessed by AI-guided quantified analysis by Heartflow^®^ identified 0 mm^3^ plaque in any vessels, placing him in the lowest percentile for atherosclerotic plaque. Conclusions: This case represents an extreme and extensively characterized example of the LMHR phenotype and highlights the limitations of extrapolating cardiovascular risk from LDL-C levels alone without consideration of broader patient context and the etiology of hypercholesterolemia. While a single case cannot redefine clinical practice, this well-characterized case is consistent with emergent literature on LMHR, and careful study of such individuals may provide valuable insights into lipid metabolism, atherosclerosis biology, and precision cardiovascular risk assessment.

## 1. Introduction

Reduction in low-density lipoprotein cholesterol (LDL-C) and apolipoprotein B (apoB) remains one of the cornerstones of cardiovascular disease risk management. Nevertheless, substantial interindividual variability exists in both susceptibility to prolonged LDL-C and apoB exposure and response to lipid-lowering pharmacotherapy. This variability is influenced by multiple factors, including genetic predisposition and past and current metabolic health status. Furthermore, the underlying etiology of a given lipid profile may modify the absolute cardiovascular risk associated with a specific LDL-C and/or apoB level.

Characterizing this individual heterogeneity is essential for advancing precision medicine, enabling identification of the most effective therapeutic strategies for individual patients and for deepening our understanding of human lipid and cholesterol metabolism. Such insights may further refine prevailing paradigms surrounding one of the leading causes of morbidity and mortality worldwide.

Herein, we describe a case involving a previously characterized [1,2,3] male in his 30s with an unusual lipid phenotype characterized by extreme elevations in LDL-C, accompanied by elevated high-density lipoprotein cholesterol (HDL-C) and low triglycerides, but otherwise excellent metabolic health. Despite maintaining a total cholesterol level as high as 705 mg/dL and an LDL-C of 574 mg/dL for 6 years and 8 months, the patient demonstrates no evidence of coronary atherosclerosis on coronary computed tomography angiography (CCTA) imaging, either by expert manual read or AI-guided quantification.

We present the case in detail, highlight its distinguishing features, and contextualize these findings within the emerging literature.

## 2. Case Description

Patient N is a male in his 30s with a medical history significant for ulcerative colitis. Despite treatment with oral and suppository mesalamine and hydrocortisone enemas, he was unable to maintain remission for longer than eight weeks. In his early 20s, he adopted an experimental ketogenic diet emphasizing seafood, extra-virgin olive oil, nuts and seeds, and low-carbohydrate vegetables, with an approximate macronutrient distribution of 80% fat (unsaturated:saturated fatty acid ratio of ~3:1), 18% protein, and 2% carbohydrate. Within two weeks of ketogenic diet initiation, the patient entered clinical remission; and a subsequent colonoscopy demonstrated biopsy-proven remission. Since commencing the ketogenic diet on June 1, 2019, he has largely and consistently maintained the dietary pattern to the present.

Since initiating the ketogenic diet, the patient has experienced colitis flares on only three occasions, each occurring during attempts to reintroduce carbohydrates in the form of honey, fruit, or starches. Otherwise, he has maintained excellent health.

After ketogenic diet initiation, the patient’s lipid profile demonstrated a marked increase in LDL-C, rising from a baseline of 95 mg/dL on a mixed macronutrient diet to 321 mg/dL, with continued escalation to a peak measured LDL-C of 574 mg/dL. This was accompanied by a total cholesterol of 705 mg/dL, HDL-C of 124 mg/dL, and triglycerides of 34 mg/dL (Table 1).

Nevertheless, over approximately seven years, with brief exceptions during brief carbohydrate reintroduction trials, he has consistently maintained total cholesterol levels between approximately 500 and 700 mg/dL and LDL-C levels between approximately 400 and 600 mg/dL. Other potentially relevant markers include apolipoprotein B levels >300 mg/dL and a consistently pattern A phenotype; lipoprotein(a) ranging from 100 to 194 nmol/L; hsCRP <1 mg/L; euthyroid with TSH values consistently between 0.5 and 1.5 mIU/L; hemoglobin A1c of ~5%; a normal comprehensive metabolic panel including transaminases; and fasting insulin levels <3 μIU/mL (Table 1).

Additionally, the patient does not have any form of congenital dyslipidemia, as demonstrated by a baseline unmedicated LDL-C level of 90–95 mg/dL on a mixed diet and confirmed by the absence of any causal variants for familial hypercholesterolemia (FH) on genome sequencing. Family history is notable for a father with a 99% occlusion of the left anterior descending artery at age 44, in the context of metabolic syndrome, obesity, and low-normal LDL-C, <70 mg/dL. On the maternal side, there is a history of hypercholesterolemia without known cardiovascular disease; notably, a CCTA at age 60 demonstrated a CAD-RADS score of 0.

With respect to pharmacotherapy, the patient has trialed statins (rosuvastatin and atorvastatin), ezetimibe, and bempedoic acid. Statin therapy (rosuvastatin) reduced LDL-C by approximately 31%; however, treatment was discontinued due to intolerance, manifested as muscle pain accompanied by elevated creatine kinase levels. Bempedoic decreased the patient’s LDL-C by 28% over six weeks, and ezetimibe decreased both the patient’s LDL-C and ApoB by 56% over six weeks.

Additional defining features of patient N’s biophysical profile include marked leanness and athleticism, with body fat consistently maintained below 12%, resting heart rate in the 40s, and a history of performing competitively in several sports including marathons. These characteristics are relevant to his lipid profile, as he meets the criteria for what has been termed a “lean mass hyper-responder” (LMHR) [4] a phenotype characterized by elevated LDL-C and HDL-C with low triglycerides in the context of persons adopting low-carbohydrate diets who are lean, generally athletic and/or with high energy demands.

Patient N represents a canonical example of this phenotype, exhibiting not only markedly elevated LDL cholesterol and apoB levels, but also pronounced lipid variability in response to lifestyle modification.

Prior published work has demonstrated reductions in LDL cholesterol and apoB levels in this phenotype with carbohydrate reintroduction, including in patient N himself. He electively demonstrated this effect in a widely reported experiment in which consumption of an additional 100 g/day of carbohydrates from commercially available Oreo cookies resulted in a 71% reduction in LDL cholesterol over 16 days [3], more than doubling the impact of high-dose statin treatment in this crossover study.

Most recently, on 3 February 2026, 6 years, 8 months and 2 days after initiating the ketogenic diet, the patient underwent CCTA imaging.

Specifically, axial prospectively ECG-triggered images were obtained using a Siemens Force Cardiac CT Scanner. Contrast-enhanced ECG-synchronized CT angiography of the heart and coronary arteries was performed, including 3D image postprocessing. Multiphase data was acquired at multiple phases of the R-R interval: mid- to late diastole. Multiplanar post-processing and 3D volume rendering were performed and interpreted. A maximum width full field of view was also reconstructed and reviewed. The 2026 images were also compared to a prior CCTA conducted on 13 October 2021, two years after patient N developed hypercholesterolemia. That prior CCTA likewise exhibited a CAD-RADS score of 0, indicating no stenosis. Similarly, no coronary plaque or stenosis was observed in any vessel in the 2026 (seven-year) scan, with a coronary artery calcium score of 0 and a CAD-RADS score of 0.

Subsequently, quantification of coronary plaque as assessed by AI-guided quantified analysis by Heartflow^®^ identified 0 mm^3^ of plaque in any vessel. HeartFlow’s AI measures coronary plaque volume from CT scans by first using deep learning to map out the coronary arteries and precisely define both the inner lumen and outer vessel walls. It then analyzes the tissue within these boundaries using CT density values to identify and classify plaque. Because each pixel in the scan represents a known 3D unit (voxel), the system labels all plaque-containing voxels and sums their volumes across the artery. This voxel-based approach allows the software to calculate total plaque burden with high precision, reporting results down to the nearest cubic millimeter (mm^3^).

**Figure 1:**
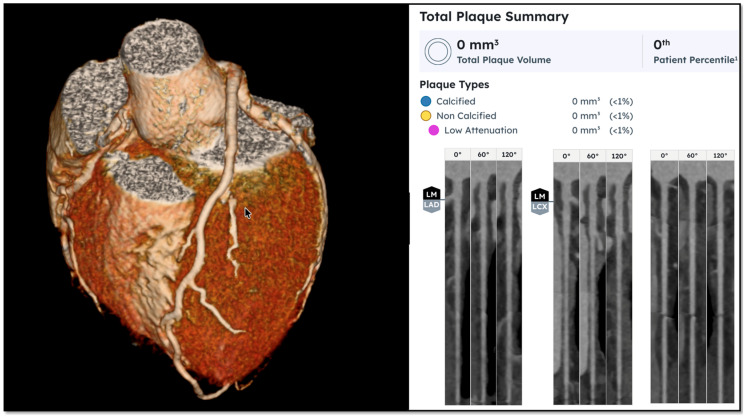
Computed tomography angiography imaging obtained of patient N’s heart, 6 years and 8 months after he commenced a ketogenic diet and developed the lean mass hyper-responder phenotype with total cholesterol over 700 mg/dL (LDL cholesterol 574 mg/dL). Quantification of atherosclerotic plaque by Heartflow^®^ AI identified 0 mm^3^ of plaque volume, placing patient N in the lowest percentile for plaque development.

HeartFlow is among the most extensively studied and clinically validated AI platforms for coronary CT analysis. Anecdotally, plaque volumes of ~20–~61.3 mm^3^ are typical for individuals in their 30s, although specific data on the reference population were not available for our review. That said, patient N’s total plaque volume of 0 mm^3^ places him, by definition, in the lowest percentile for atherosclerotic plaque (Figure 1).

## 3. Discussion

This case is certainly exceptional, although not entirely unique. Over the past several years, the growing body of literature has described the LMHR phenotype, including testable hypotheses regarding the physiological basis for the combination of markedly elevated LDL-C alongside otherwise favorable lipid features—namely high HDL-C and low triglycerides [5]—as well as its associated cardiovascular risk profile [6].

Most recently, a study following 100 adults with this phenotype adhering to ketogenic diets (as confirmed by daily beta-hydroxybutyrate measurements and dietary logs) reported variable but generally minimal relevant plaque progression, as assessed by AI–guided coronary CT angiography measurements (QAngio^®^ and HeartFlow^®^) [6]. A substantial proportion of participants exhibited no plaque progression, and some demonstrated confirmed biological regression.

Importantly, across all AI-guided CTA and geographic plaque measurements in this cohort of 100 LMHR and near-LMHR patients, similar to patient N, neither LDL-C exposure nor apoB levels predicted plaque progression, despite the cohort exhibiting the largest LDL and apoB spread ever recorded in a prospective cardiac imaging study.

Instead, baseline plaque burden was the primary predictor of subsequent progression, suggesting either an underlying susceptibility to atherosclerosis or a localized biological amplification process in which existing plaque promotes further plaque development.

Patient N represents an extreme manifestation of this phenotype, having maintained LDL-C levels near the upper bound of those observed in LMHR cohorts for nearly seven years and being among the most extensively characterized individuals described to date.

The prevailing explanation for this phenotype is the lipid energy model, which posits that in lean individuals consuming very low-carbohydrate diets, hepatic very-low-density lipoprotein (VLDL) synthesis and export are upregulated to meet whole-body energy demands in a metabolic environment characterized by high rates of fat oxidation and low circulating insulin. Accelerated lipoprotein lipase (LPL)-mediated triglyceride clearance in adipose and skeletal muscle tissue leads to depletion of circulating triglycerides and the generation of LDL particles and increased HDL-C as downstream products, producing the characteristic triad of very high LDL-C, very high HDL-C, and low triglycerides observed in patient N and other LMHRs.

Importantly, the lipid energy model [7] proposes testable hypotheses about the variables that modulate LDL-C levels in LMHRs. In patient N, increases in carbohydrate intake, modest weight gain, and reductions in energy expenditure have each been associated with substantial LDL-C reductions, whereas increases in aerobic activity reliably produce LDL-C elevations. These effects have been replicated in others with the LMHR phenotype.

How the lipid energy model, and other physiologic explanations for the LMHR phenotype, translate into an altered risk profile remains an open question. Possibilities include differences in lipoprotein flux and clearance, particle characteristics (size, density, and associated apolipoproteins), overall metabolic health, and hormonal mediators such as adiponectin [8], FGF-21 [9,10,11], and ANGPTLs [12,13,14]. Future studies should aim to clarify which of these or other protective factors might mediate the apparent resistance of N and similar patients to the development of atherosclerosis despite extreme hypercholesterolemia.

Patient N’s decision to remain largely unmedicated between the time of his diagnosis with extreme hypercholesterolemia and his recent CCTA scan merits particular consideration given his scientific literacy as an MD-PhD with expertise in human metabolism and lipidology.

While the prevailing consensus supports lipid-lowering therapy for cardiovascular risk reduction, outcome data are derived from populations with insulin resistance, excess adiposity, or congenital lipid disorders such as FH. It is worth highlighting that the pathophysiology of FH—particularly homozygous FH—is fundamentally distinct from the reversible, diet-induced metabolic adaptation observed in LMHRs, despite inducing similar LDL-C levels.

Whether LMHRs share comparable cardiovascular risk remains understudied, but the available phenotype-specific evidence suggests they may not (discussed above). Notably, patient N’s elevated Lp(a) levels (100–194 nmol/L) represent an independent, genetically determined cardiovascular risk factor that, unlike his LDL-C and ApoB, are not dramatically modified by lifestyle [15]. If anything, this makes N’s case even more impressive and raises open questions about the atherogenicity of this other lipoprotein particle, Lp(a), in the context of good metabolic health. Indeed, some recent evidence suggests that variables such as low abdominal adiposity, as indicated by a low waist-to-hip ratio, may reduce or eliminate cardiovascular risk associated with high Lp(a) [16].

In general, there is a paucity of evidence on the absolute risks associated with assumed-to-be atherogenic apoB-associated lipoproteins (LDL and Lp(a)) in otherwise metabolically healthy individuals without underlying genetic disorders of lipid metabolism. Further studies of such populations, including LMHRs like patient N, would provide greater insight into the intrinsic atherogenicity (or lack thereof) of these particles.

For patient N, concerns regarding statin therapy include effects on insulin sensitivity, diabetes risk, mitochondrial toxicity [17], reductions in GLP-1 levels [18], and potential loss of lean muscle mass over time, particularly in active individuals like himself [19]. He is, however, still considering alternative agents, specifically ezetimibe and bempedoic acid, given early and emerging data on potential benefits beyond lipid management including possible neuroprotective and broader metabolic health effects [20,21]. With respect to ezetimibe, after completion of the CCTA scan presented in his report, he recommenced a trial of 10 mg ezetimibe (starting at 5 mg once per day and titrating up to 10 mg twice per day with food to help with gastrointestinal tolerance). As noted above, his response to this ezetimibe monotherapy included 56% reductions in LDL-C and ApoB. If he continues to be devoid of side-effects, and pending new data that may update his opinion, he attests he is likely to remain on ezetimibe primarily for the potential neuroprotective benefits [21].

Ultimately, patient N, and others like him who are sometimes accused of being “non-compliant” or “irresponsible,” are not categorically opposed to pharmacologic therapy but are navigating treatment decisions in the context of profound evidentiary gaps. This case underscores the enduring reality of clinical medicine: important decisions must often be made with incomplete data. How the biomedical community responds to such gaps will shape both patient care and future understanding of lipid metabolism and cardiovascular risk.

## 4. Conclusions

Despite living largely unmedicated—and for nearly seven years with a total cholesterol around 700 mg/dL—patient N exhibits no detectable atherosclerotic plaque, including 0 mm^3^ of plaque volume on AI-guided analysis of his CCTA heart scan, placing him in the lowest percentile for total plaque. Patient N represents an example of an emerging phenotype: the lean mass hyper-responder. Individuals with this phenotype exhibit striking and dynamic changes in lipid levels in response to carbohydrate restriction, likely reflecting adaptive metabolic shifts that help meet energy demands. Given the unprecedented nature of this phenotype and its underlying physiology, one might expect risk profiles that diverge from conventional expectations. In this case, that is clearly what we observe. It is a humbling reminder of how much we still have to learn about human lipidology, and an inspiration for much-needed further research.

## 5. Patient Perspective

When I first discovered my hypercholesterolemia, I was terrified. It felt like a death sentence at the time, and I felt trapped between a rock and a hard place: adhering to the first intervention—in this case, a ketogenic diet—that worked for my ulcerative colitis and brought back my quality of life, and a lipid profile that would almost certainly kill me.

However, alongside the fear was a growing curiosity about why my cholesterol levels had increased more than sixfold. It didn’t resemble anything typical or previously described that I could find. When I discovered that there are many others like me—lean mass hyper-responders who adopt ketogenic diets for therapeutic reasons and experience dramatic increases in LDL—and that we tend to share traits such as being lean, active, metabolically healthy, and insulin sensitive, I became fascinated. Still concerned, but deeply intrigued.

I believe we represent a group of outliers who remain understudied, yet offer an extraordinary opportunity for scientific discovery. Ours is a kind of natural experiment—one that isolates high LDL in the absence of other cardiovascular risk factors or a clear genetic cause. We deserve to be studied, not only for personal reasons or to guide individual care, but for what we might reveal more broadly about human health. And I suspect we are far less rare than most people assume.

## Figures and Tables

**Table 1:** Baseline markers were obtained from May 2019, shortly before N adopted a ketogenic diet. Current measurements reflect the most recent unmedicated values.

	Total Cho. mg/dL	LDL-C mg/dL	HDL-C mg/dL	Triglycerides mg/dL	ApoB mg/dL	Lp(a) nmol/L	hsCRP mg/L	Insulin uIU/mL
**Baseline**	160	95	48	76	71	109	1.0	<3
**Present**	705	574	124	34	335	194	0.3	<3
**Coronary Computed Tomography Angiography Imaging**								
CAC = 0								
CAD-RADS = 0								
Total plaque volume = 0 mm^3^								

## Data Availability

Data inquiries may be directed to the corresponding author.

## References

[1] Norwitz N.G., Loh V. (2020). A standard lipid panel is insufficient for the care of a patient on a high-fat, low-carbohydrate ketogenic diet. Front. Med..

[2] Norwitz N.G., Soto-Mota A., Feldman D., Parpos S., Budoff M. (2022). Case Report: Hypercholesterolemia “Lean Mass Hyper-Responder” Phenotype Presents in the Context of a Low Saturated Fat Carbohydrate-Restricted Diet. Front. Endocrinol..

[3] Norwitz N.G., Cromwell W.C. (2024). Oreo Cookie Treatment Lowers LDL Cholesterol More Than High-Intensity Statin therapy in a Lean Mass Hyper-Responder on a Ketogenic Diet: A Curious Crossover Experiment. Metabolites.

[4] Norwitz N.G., Feldman D., Soto-Mota A., Kalayjian T., Ludwig D.S. (2021). Elevated LDL-cholesterol with a carbohydrate-restricted diet: Evidence for a ‘lean mass hyper-responder’ phenotype. Curr. Dev. Nutr..

[5] Norwitz N.G., Soto-Mota A., Kaplan B., Ludwig D.S., Budoff M., Kontush A., Feldman D. (2022). The Lipid Energy Model: Reimagining Lipoprotein Function in the Context of Carbohydrate-Restricted Diets. Metabolites.

[6] Budoff M., Kinninger A., Manubolu V.S., Norwitz N.G., Feldman D., Soto-Mota A. (2026). The Impact of Sustained LDL-C Elevation on Plaque Changes: Primary Coronary plaque progression results from the Keto CTA Study. medRxiv.

[7] Norwitz N.G., Mindrum M.R., Giral P., Kontush A., Soto-Mota A., Wood T.R., D’AGostino D.P., Manubolu V.S., Budoff M., Krauss R.M. (2022). Elevated LDL-cholesterol levels among lean mass hyper-responders on low-carbohydrate ketogenic diets deserve urgent clinical attention and further research. J. Clin. Lipidol..

[8] Terazawa-Watanabe M., Tsuboi A., Fukuo K., Kazumi T. (2014). Association of adiponectin with serum preheparin lipoprotein lipase mass in women independent of fat mass and distribution, insulin resistance, and inflammation. Metab. Syndr. Relat. Disord..

[9] Szczepanska E., Gietka-Czernel M. (2022). FGF21: A Novel Regulator of Glucose and Lipid Metabolism and Whole-Body Energy Balance. Horm. Metab. Res..

[10] Domouzoglou E.M., Maratos-Flier E. (2011). Fibroblast growth factor 21 is a metabolic regulator that plays a role in the adaptation to ketosis. Am. J. Clin. Nutr..

[11] Fisher F.M., Chui P.C., Antonellis P.J., Bina H.A., Kharitonenkov A., Flier J.S., Maratos-Flier E. (2010). Obesity is a fibroblast growth factor 21 (FGF21)-resistant state. Diabetes.

[12] Abu-Farha M., Al-Khairi I., Cherian P., Chandy B., Sriraman D., Alhubail A., Al-Refaei F., AlTerki A., Abubaker J. (2016). Increased ANGPTL3, 4 and ANGPTL8/betatrophin expression levels in obesity and T2D. Lipids Health Dis..

[13] Aryal B., Price N.L., Suarez Y., Fernandez-Hernando C. (2019). ANGPTL4 in Metabolic and Cardiovascular Disease. Trends Mol. Med..

[14] Catoire M., Alex S., Paraskevopulos N., Mattijssen F., Evers-van Gogh I., Schaart G., Jeppesen J., Kneppers A., Mensink M., Voshol P.J. (2014). Fatty acid-inducible ANGPTL4 governs lipid metabolic response to exercise. Proc. Natl. Acad. Sci. USA.

[15] Reyes-Soffer G., Ginsberg H.N., Berglund L., Duell P.B., Heffron S.P., Kamstrup P.R., Lloyd-Jones D.M., Marcovina S.M., Yeang C., Koschinsky M.L. (2022). Lipoprotein(a): A Genetically Determined, Causal, and Prevalent Risk Factor for Atherosclerotic Cardiovascular Disease: A Scientific Statement From the American Heart Association. Arterioscler. Thromb. Vasc. Biol..

[16] Ahmad M.I., Chevli P.A., Mirzai S., Rikhi R., Bhatia H., Pagidipati N., Blumenthal R., Razavi A.C., Ruddiman K., Spitz J.A. (2025). Waist to hip ratio modifies the cardiovascular risk of lipoprotein(a): Insights from MESA. Prog. Cardiovasc. Dis..

[17] Ryan T.E., Torres M.J., Lin C.T., Clark A.H., Brophy P.M., Smith C.A., Smith C.D., Morris E.M., Thyfault J.P., Neufer P.D. (2024). High-dose atorvastatin therapy progressively decreases skeletal muscle mitochondrial respiratory capacity in humans. JCI Insight.

[18] She J., Tuerhongjiang G., Guo M., Liu J., Hao X., Guo L., Liu N., Xi W., Zheng T., Du B. (2024). Statins aggravate insulin resistance through reduced blood glucagon-like peptide-1 levels in a microbiota-dependent manner. Cell Metab..

[19] Gentreau M., Sakr M., Mohammad S., Alsehli A.M., Titova O.E., Rukh G., Schioth H.B. (2025). Statin Use Is Associated With a Decline in Muscle Function and Mass Over Time, Irrespective of Statin Pharmacogenomic Score. J. Cachexia Sarcopenia Muscle.

[20] Papa C., Rose A., Martin H.N.G., Useini A., Geier F., Liao L., Rodriguez-Aguilera J.R., Valina-Allo P., Hoffmann A., Tvardovskiy A. (2026). Bempedoic acid directly binds and activates PPARalpha. Cell Metab..

[21] Ganne A., Mainali N., Balasubramaniam M., Atluri R., Pahal S., Asante J., Nagel C., Vallurupalli S., Shmookler Reis R.J., Ayyadevara S. (2024). Ezetimibe Lowers Risk of Alzheimer’s and Related Dementias over Sevenfold, Reducing Aggregation in Model Systems by Inhibiting 14-3-3G::Hexokinase Interaction. Aging Biol..

